# Percutaneous Edge-to-Edge Valve Interventions: The Role of Surgical Salvage in Complex Percutaneous Techniques

**DOI:** 10.7759/cureus.60938

**Published:** 2024-05-23

**Authors:** Mahati Dasari, Pramukh Arun Kumar, Zeynep Yukselen, Pradnya Brijmohan Bhattad, Mark Kranis, Joseph Hannan

**Affiliations:** 1 Internal Medicine, Saint Vincent Hospital, Worcester, USA; 2 Cardiovascular Medicine, Saint Vincent Hospital, UMass Chan Medical School, Worcester, USA; 3 Cardiovascular Medicine, Saint Vincent Hospital, Worcester, USA

**Keywords:** transcatheter mitral valve edge-to-edge repair, mitraclip, mitral valve regurgitation, redo-mitraclip, mitral valve replacement

## Abstract

Hemodynamically significant mitral regurgitation (MR) is associated with major morbidity and mortality. Transcatheter edge-to-edge repair (TEER) is an interventional procedure for MR, which has gained popularity in recent years as an alternative solution to surgical valve repair in high-risk surgical candidates. However, there are no definite guidelines following TEER failures to determine if patients would benefit from a redo TEER or surgical mitral valve (MV) repair. Here, we present one such clinical dilemma.

In patients who have failed the TEER of the MV, surgical risk must be determined in conjunction with a multidisciplinary team, as surgical MV replacement may be performed at advanced centers in high-risk patients with good results.

## Introduction

Transcatheter edge-to-edge repair (TEER) is an interventional procedure for mitral regurgitation (MR) in high-risk surgical patients that offers acceptable short-term results as well as improved survival and quality of life in long-term follow-up studies [1].

The MitraClip (Abbott, Santa Clara, CA) is a transcatheter technology based on the surgical Alfieri edge-to-edge repair creating a "double orifice" mitral regurgitant area [1]. The MitraClip system utilizes a cobalt-chromium clip covered with a polypropylene fabric that grasps the mitral valve (MV) leaflets, reducing MR by increasing the coaptation between the regurgitant valve leaflets. In some cases, a second clip may be required to adequately reduce the MR severity toward a goal of final regurgitant severity ≤2+ [2-4].

MV repair may not be an option for most patients with MitraClip failure due to severe injury to the leaflets resulting from clip implantation causing local tissue destruction and fibrosis, particularly redo MitraClip failure [2,3]. For such patients, MV replacement may be the only reasonable option, accepting a high perioperative risk and comparative lower durability to valve repair. Here, we present a case of redo MitraClip failure managed with surgical MV replacement [4,5].

## Case presentation

We present a case of a 74-year-old female with a past medical history of hypertension, hyperlipidemia, persistent atrial fibrillation (AFib), advanced chronic obstructive pulmonary disease, severe pulmonary hypertension, heart failure with preserved ejection fraction (EF), and severe primary MR due to myxomatous anterior MV prolapse who is status post two separate MitraClip procedures with a total placement of three clips. She initially had a MitraClip NTR placement and a redo MitraClip placement with two clips, one MitraClip G4 NTW and one MitraClip G4 NT, for single leaflet detachment 10 months later due to recurrent severe MR as a result of loss of posterior leaflet insertion. Fifteen months after the redo procedure, she presented to her primary care physician with worsening dyspnea on exertion of four months duration associated with progressive lower extremity swelling. She was prescribed increasing doses of diuretics without symptomatic improvement and subsequently presented to the emergency room with worsening symptoms and extreme fatigue. Her dyspnea had progressed from New York Heart Association (NYHA) class II to NYHA class III-IV and affected her activities of daily living. She also reported associated orthopnea and paroxysmal nocturnal dyspnea. She denied chest pain, chest tightness, palpitations, syncope, fever, chills, cough, or sputum production. At the time of presentation, she saturated at 95% on 4 liters via nasal cannula with blood pressures in the 80s/50s. She was also tachycardic to the 110s with an irregular heart rhythm. A holosystolic murmur and a displaced apical impulse were heard, maximally at the apex. A respiratory exam revealed diminished breath sounds with inspiratory and expiratory crackles heard in the bibasilar lung fields. She also had 3+ pitting pedal edema bilaterally in her lower extremities extending to her knees. She was hospitalized for further investigations and management.

**Figure 1:**
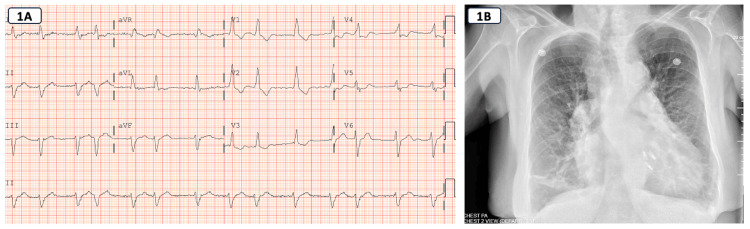
(A) ECG showing atrial fibrillation. (B) Chest X-ray demonstrating findings of pulmonary venous hypertension with interstitial edema and cardiomegaly. MitraClips can be visualized as well.

Standard laboratory tests were normal except for elevated serum bicarbonate at 40 (reference range: 22-29 mEq/L) and a pro-B-type natriuretic peptide that was abnormal at 7000 pg/mL (reference range: 450 pg/mL). A chest X-ray revealed cardiomegaly, findings of pulmonary arterial hypertension, as well as findings of pulmonary venous hypertension in the form of upper lobe veins more prominent than lower lobe and increased interstitial markings (Figure 1). An electrocardiogram showed AFib with no evidence of ischemic ST-T changes (Figure 1).

A transthoracic echocardiogram (TTE) revealed mild concentric left ventricular hypertrophy (LVH) and normal left ventricular systolic function with an EF of 65%. The right ventricle was enlarged with normal right ventricular systolic function, and a flattened interventricular septum consistent with right ventricular pressure/volume overload was noted. Multiple mitral clips were seen attached to MV leaflets, and an eccentric MR jet was present (MV E/A: 3.3, MR max velocity: 391.7 cm/sec, and MR max peak gradient: 61.4 mm of Hg); however, it was difficult to assess the severity of the MR. At admission, her diuretics were initially withheld due to contraction alkalosis; however, soon thereafter, they were resumed due to worsening volume overload and oxygen requirement.

A transesophageal echocardiogram showed severe MR with malcoaptation of the MV leaflets. The regurgitant jet was eccentric and posteriorly directed. The effective regurgitant orifice area (EROA) and regurgitant volume (RVol) were calculated using the proximal isovelocity surface area (PISA) method, as the continuity method was deemed inappropriate in the setting of AFib. The EROA was 47 mm^2^, with an RVol of 68 mL, a volume flow rate of 293 mL/s, and a vena contracta of 0.7 cm, all consistent with severe MR. Three distinct MitraClips attached to the MV apparatus were seen. The left atrium was severely dilated with a left atrial volume index of 52 mL/m2, and the left ventricular EF was normal with no regional wall motion abnormalities.

**Figure 2:**
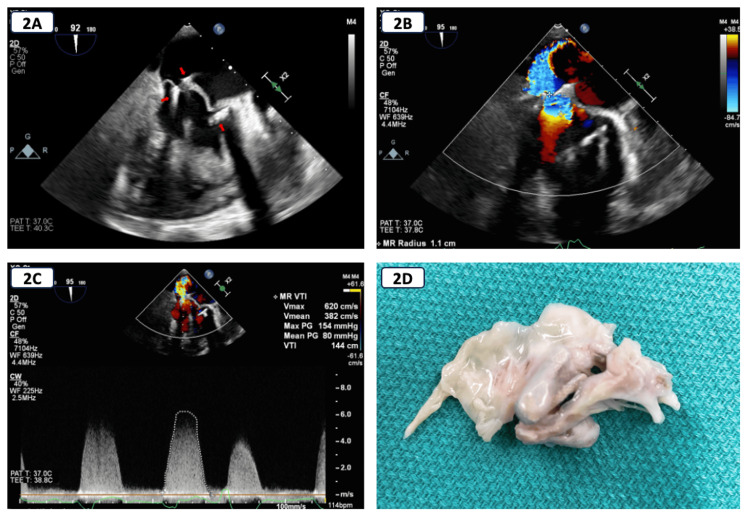
Transesophageal echocardiogram and gross pathology. A: Transesophageal echocardiogram showing mitral apparatus with three MitraClips (arrowheads). B: Transesophageal echocardiogram showing eccentric posterolaterally directed mitral regurgitation (MR) jet. C: Transesophageal echocardiogram showing MR Doppler trace. D: Resected native mitral valve with MitraClips.

For recurrent severe MR resulting in decompensated heart failure, definitive therapy necessitated treatment of the MR. Options included another redo MitraClip procedure versus surgical MV replacement. After extensive discussion with a multidisciplinary heart valve team and the patient, another redo MitraClip procedure was deemed inappropriate due to high periprocedural risk and a lack of a target implantation site for another clip. Hence, a decision was made to proceed with surgical MV replacement accepting a high operative risk with a Society of Thoracic Surgeons (STS) risk score of >8%. During surgery, the originally placed MitraClip appeared to have inverted on the anterior leaflet with no posterior leaflet attachment. The other two clips had very narrow tissue bridges and had densely scarred into the small, thickened posterior leaflet leaving an unrecognizable MV remaining. The anterior leaflet was excised to the annulus, and the posterior leaflet had to be excised at the annulus for most of its attachment due to the extensive destruction by the clips. There was one residual heavily calcified cord that was also excised. A 29 mm Epic plus bioprosthetic porcine valve was placed successfully (Figure 2).

The patient was admitted to the critical care unit for postoperative care. The postoperative course was complicated by escalating vasopressor requirement, up to a maximum of four agents, including epinephrine, norepinephrine, milrinone, and vasopressin, which were weaned off in three days. She also had severe pulmonary hypertension, which led to difficulty weaning from the ventilator. She was briefly placed on epoprostenol and was transitioned to sildenafil. Repeat TTE on postoperative day two showed a normal EF, mild concentric LVH, and well-seated bioprosthetic MV with no MR. Her hypotension was likely secondary to acute blood loss during the surgery, for which she required three units of packed red blood cells. Although she had a prolonged postoperative course in the hospital, her clinical status significantly improved, and she was successfully discharged to a short-term rehabilitation center and eventually home with her baseline oxygen support and resolution of presenting symptoms. At one year follow-up, the patient has been doing well with minimal supplemental oxygen requirement.

## Discussion

Patients undergoing evaluation for TEER should be evaluated by a multidisciplinary heart team that includes a structural interventional cardiologist, an imaging physician, a heart failure specialist, and a cardiac surgeon. Prohibitive surgical risk is based on a 30-day STS-predicted operative mortality of ≥8% for MV replacement [6,7]. However, STS estimated risk does not capture all relevant risk factors, so it should be combined with other risk assessments [8]. Our patient was deemed high risk for surgery given multiple comorbidities and an STS score of >8%, hence had a MitraClip procedure performed.

However, our patient failed the MitraClip procedure and had recurrent symptomatic MR. There are several causes of MitraClip failure, which include single leaflet detachment, loss of leaflet insertion, and clip embolization [9]. In the setting of a failed MitraClip procedure, depending on the circumstances and duration of clip implantation, significant destruction and fibrosis of the leaflets may occur, precluding surgical MV repair [2]. A retrospective study using international registry data between 2009 and 2020 showed better morbidity and mortality outcomes with redo MitraClip than surgical or conservative management [9]. Hence, a redo MitraClip placement was opted for our patient.

Despite these interventions, she developed progressive heart failure symptoms secondary to recurrent MR after 15 months. There is minimal literature on further managing redo MitraClip failure with surgical MV repair or replacement. Data show that assessment of surgical risk determined in conjunction with a multidisciplinary team as MV repair is frequently performed at advanced centers on high-risk patients with good results [10]. Since our patient had malcoaptation of the MV leaflets on imaging, a multidisciplinary team decided to proceed with surgical MV replacement, despite the high surgical risk.

## Conclusions

Surgical MV replacement might be the only reasonable option for patients who have failed the redo MitraClip procedure associated with significant destruction and fibrosis of the leaflet leaves. However, surgical risk determined in conjunction with a multidisciplinary team approach and involving the patient in shared decision-making can be helpful in these patients providing good results as seen with our patient.
